# Social Network Analysis of the Effects of a Social Media–Based Weight Loss Intervention Targeting Adults of Low Socioeconomic Status: Single-Arm Intervention Trial

**DOI:** 10.2196/24690

**Published:** 2021-04-09

**Authors:** Ran Xu, David Cavallo

**Affiliations:** 1 Department of Allied Health Sciences College of Agriculture, Health and Natural Resources University of Connecticut Storrs, CT United States; 2 Department of Nutrition School of Medicine Case Western Reserve University Cleveland, OH United States

**Keywords:** weight loss intervention, social media intervention, electronic health, social network analysis

## Abstract

**Background:**

Obesity is a known risk factor for cardiovascular disease risk factors, including hypertension and type II diabetes. Although numerous weight loss interventions have demonstrated efficacy, there is considerably less evidence about the theoretical mechanisms through which they work. Delivering lifestyle behavior change interventions via social media provides unique opportunities for understanding mechanisms of intervention effects. Server data collected directly from web-based platforms can provide detailed, real-time behavioral information over the course of intervention programs that can be used to understand how interventions work.

**Objective:**

The objective of this study was to demonstrate how social network analysis can facilitate our understanding of the mechanisms underlying a social media–based weight loss intervention.

**Methods:**

We performed secondary analysis by using data from a pilot study that delivered a dietary and physical activity intervention to a group of participants via Facebook. We mapped out participants’ interaction networks over the 12-week intervention period and linked participants’ network characteristics (eg, in-degree, out-degree, network constraint) to participants’ changes in theoretical mediators (ie, dietary knowledge, perceived social support, self-efficacy) and weight loss by using regression analysis. We also performed mediation analyses to explore how the effects of social network measures on weight loss could be mediated by the aforementioned theoretical mediators.

**Results:**

In this analysis, 47 participants from 2 waves completed the study and were included. We found that increases in the number of posts, comments, and reactions significantly predicted weight loss (β=–.94, *P*=.04); receiving comments positively predicted changes in self-efficacy (β=7.81, *P*=.009), and the degree to which one’s network neighbors are tightly connected with each other weakly predicted changes in perceived social support (β=7.70, *P*=.08). In addition, change in self-efficacy mediated the relationship between receiving comments and weight loss (β=–.89, *P*=.02).

**Conclusions:**

Our analyses using data from this pilot study linked participants’ network characteristics with changes in several important study outcomes of interest such as self-efficacy, social support, and weight. Our results point to the potential of using social network analysis to understand the social processes and mechanisms through which web-based behavioral interventions affect participants’ psychological and behavioral outcomes. Future studies are warranted to validate our results and to further explore the relationship between network dynamics and study outcomes in similar and larger trials.

## Introduction

Obesity is a common disease with a high economic burden, and it is a known risk factor for several chronic diseases, including cancer, cardiovascular diseases, and diabetes [1]. Individuals with low socioeconomic status (SES) are disproportionately more likely to be obese and develop related diseases [2,3]. While there are a vast number of behavioral weight loss interventions established in previous literature [4], relatively few studies of high quality have targeted participants of low SES. There is some evidence that groups with low SES experience lower efficacy in weight loss interventions, especially those that focus only on individual-level determinants [5-7]. This highlights the need for the development of effective behavioral weight loss strategies for individuals with low SES.

A potentially effective way of delivering behavioral weight loss interventions to participants of low SES is the use of social media platforms (eg, Facebook, Twitter). Social media is widely used by internet users of low SES [8]. Compared with face-to-face interventions, web-based delivery of social media offers advantages such as continuous availability and remote access, which could reduce barriers to intervention use among participants of low SES, such as limited time or inadequate transportation [9,10]. In addition, social media users are accustomed to sharing information about their health experiences and opinions [11,12]. This sharing may be enhanced by the communication features of social media websites, which can facilitate sharing and communication among users about weight loss behaviors, goals, and experiences [13,14]. These features make social media platforms particularly attractive for delivering behavioral weight loss interventions. Indeed, some preliminary evidence suggests that social media–based interventions can be efficacious in producing weight loss and increasing the frequency of social interaction and social support [15]. Results from a handful of social media–based behavioral weight loss interventions targeting participants of low SES show that participants have similar receptiveness to the intervention format and weight loss outcome when comparing in-person and social media intervention delivery [16,17]. However, as the evidence of the efficacy of social media–based weight loss intervention accumulates, our understanding of the mechanisms underlying social media–based weight loss interventions is still somewhat limited. Previous literature suggests that constructs such as social support and social comparison are important weight loss predictors. Some studies have linked social media–based intervention components with increased social support, positive behavior change, and health outcomes [18,19]. Studies have also reported greater weight loss for participants in teams or those reporting greater social influence [20]. This evidence, however, is scattered in the context of social media–based weight loss interventions and is largely based on cross-sectional or post-hoc studies. The mechanisms through which social media–based weight loss interventions change participants’ interactions, theoretical mediators (eg, knowledge, social support, self-efficacy), and subsequent weight loss behavior and outcomes are still largely unclear.

A promising approach to increase our understanding of the mechanisms underlying social media–based weight loss interventions is to study the user-generated content and interactions during the intervention by using social network analysis. Previous research has shown that interactions generated through social networks serve as important sources for information and knowledge [21-23], social and physical resources [24,25], and social support [26,27]. In addition, social networks influence individual behavior and perception through various mechanisms such as sensemaking, norms, and learning [28,29]. Thus, it is possible to leverage social network analysis to better understand users’ position and role in the interaction networks and how that affects the theoretical mediators and subsequent weight loss behavior and outcomes.

In this study, using data from a 12-week pilot behavioral intervention that assessed the feasibility of a weight loss program delivered via social media to adults of low SES [30], we mapped out participants’ interaction networks and conducted social network analysis to assess how participants’ network characteristics were associated with various psychological and behavioral study outcomes, as well as the possible mediating mechanisms that lead to participants’ weight loss.

## Methods

### Design

We conducted secondary analysis of data from the INSHAPE CLE study. INSHAPE CLE was a single-arm, pre-post intervention trial designed to assess the feasibility of delivering a social media–based weight loss intervention to low-income residents of a large midwestern urban area of the United States of America. INSHAPE CLE delivered a 12-week behavioral weight loss intervention that included goal setting and self-monitoring using Fitbit devices and the Fitbit self-monitoring platform. Participants were also enrolled in a private Facebook group moderated by study personnel (Moderator), who posted planned communications to the group that delivered nutrition and physical activity education and encouraged participants to exchange social support and model desired behaviors. Moderators also responded to participant questions and monitored the group for inappropriate communications. All procedures performed in this study involving human participants were in accordance with the ethical standards of the institutional and national research committee and were approved by the Case Western Reserve University Institutional Review Board and with the 1964 Helsinki declaration and its later amendments or comparable ethical standards [31-34]. This paper does not contain any studies with animals performed by any of the authors.

### Participants

INSHAPE CLE participants were recruited using social media advertisements, flyers posted in strategic locations, and in-person recruitment events at federally sponsored nutrition education events. Primary inclusion criteria were age of 35-65 years, regular internet use, BMI of 25-40, less than 30 minutes per day of moderate or vigorous physical activity, residence in the target metropolitan area, income less than 185% of the federal poverty based on family size, and no affirmative answers on the Physical Activity Readiness Questionnaire [35] or written clearance by a physician. Participants were recruited in 2 waves and enrolled in 2 separate Facebook groups with largely identical moderator content.

### Study Procedures

Participants were screened online. Eligible participants were provided a web-based consent form and those who accepted were provided a web-based baseline questionnaire and invited to an in-person orientation meeting where study instructions were provided and anthropometric measures were collected. Participants attending an orientation meeting were considered enrolled in the study. Enrollees were provided a web-based follow-up questionnaire at the end of the 12-week intervention and invited to a meeting where follow-up anthropometric measures were taken. Additional details of the INSHAPE CLE intervention and study procedures can be found elsewhere [30].

### Measures

#### Demographics

As a part of the baseline questionnaire, participants were asked to report their birth year, gender, race, ethnicity, educational status, and employment status.

#### Anthropometrics

All anthropometrics were collected by study staff who attended a 1-hour training session. We measured height by using a stadiometer (Detecto PHR Portable Mechanical Height Rod for DR400C) and weight by using a floor scale (Detecto DR550C) calibrated using a 10-kg weight (Ohaus 80850302). Measurement procedures were based on the National Health and Nutrition Examination Survey Anthropometry Procedures Manual.

#### Theoretical Mediators

Several psychological and behavioral constructs were theorized to be important determinants of the weight loss outcome. Specifically, dietary knowledge was assessed by the sum of correct answers from a 36-item questionnaire adapted from a validated nutrition knowledge questionnaire and was scored as the percentage of questions answered correctly. The questionnaire assessed several domains of nutrition knowledge, including national dietary recommendations, the nutrient content of foods, and the relationship between dietary behavior and chronic disease. Perceived social support was measured using the 5-item Friend Social Support and Eating Habits Scale that asks participants to indicate how often their friends (1=never, 5=very often) communicated positive messages about dietary behavior (eg, discussed my eating habit changes with me [asked me how I’m doing with my eating changes]). Self-efficacy was assessed using a validated 4-item scale measure. This scale asked participants “How confident are you that you can lose weight?” with a series of conditional statements (eg, Even if you have to try several times until it works) rated on a sliding scale from 0% (not at all confident) to 100% (completely confident).

#### Social Network/Engagement Measures

**Figure 1:**
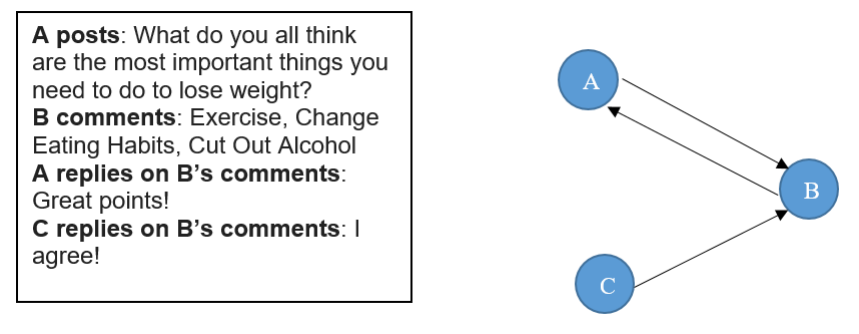
A simple example illustrating the network construction process, wherein the link represents the comment/reply relationship.

Each post, comment, and reaction (eg, emojis) on the Facebook group during the intervention was recorded. Two data collection methods were used to obtain Facebook data. The Grytics analytical platform [36] provided data directly from the Facebook application programming interface. We also collected select data manually by reviewing and logging information directly from the Facebook group. This was required due to changes to Facebook’s privacy policy that occurred during the second group’s intervention that deidentified individual Facebook user data. However, the Grytics platform did not provide data on the reactions for comments with the exception of likes. Data were collected in both ways and validated by comparing aggregate data provided by Grytics, where available, to the totals from the manual data collection. In cases where totals did not agree, Facebook was manually checked a second time to ensure that the data collection methods produce consistent data across the entire intervention period. To obtain a more nuanced understanding of subjects’ different levels/types of participation and engagement in the intervention, a social network was constructed using the comment relationship. A network tie from i to j represents i has sent out a comment/reply to j’s post or comment, with the weight representing the number of comments across the whole intervention. To further illustrate this, in the simple example shown in Figure 1, the communication stream on the left can be represented as the network on the right.

We derived several centrality measures from the social network to indicate the type or level of treatment an individual received during the intervention. Specifically, in-degree was calculated as the total number of comments each individual received during the intervention. Network constraint was calculated to represent the degree to which one’s interaction partners also interact with each other. Previous studies have linked network constraint to individual’s social capital, social support, and access to diverse information [24]. The interaction partners/network neighbors of an individual with higher network constraint are more tightly connected with each other, while the interaction partners/network neighbors of an individual with lower network constraint are more disconnected from each other. Finally, we calculated out-degree to represent participants’ engagement. As the network constructed excluded posts and reactions that one created, instead of out-degree based on the constructed network, we calculated out-degree as the sum of posts, reactions, and comments for each individual. As out-degree and in-degree are highly skewed, we used the log-transformed variables in the final analysis.

### Data Analyses

Data analyses were completed using STATA 16.0 (StataCorp). Data were screened for deviations from assumptions required for the statistical analyses used. Analyses were performed on participants who completed baseline and follow-up measures on variables used in the analysis (ie, study completers). There were no significant differences in the demographic characteristics between completers (N=47) and those lost to follow-up (n=8). We calculated descriptive statistics for baseline characteristics, outcome, and theoretical mediator measures, as well as social network measures. To examine how social network measures were associated with the change in outcome and theoretical mediators, we used multivariate regression analysis with the change in weight, dietary knowledge, social support, and self-efficacy as the outcome (ie, difference between the outcome after the intervention and at baseline) and each aforementioned social network measure as the predictor in separate regression models. In each regression analysis, we controlled for the baseline value of the outcome, the treatment group indicator (group 1 or group 2) as well as other baseline characteristics such as age, race, education status, and BMI. We reported robust standard error to account for possible heteroscedasticity. In addition, to rule out the possible bias of network construction in our results, we also performed 2 sensitivity analyses: (1) we calculated out-degree as the total number of comments each individual made during the intervention and reran the aforementioned analyses and (2) we constructed the comment networks and associated network measures by excluding moderator-related interactions (ie, participant-only network) and reran the aforementioned analysis. Detailed results are reported in Multimedia Appendix 1. Furthermore, while the original intervention was not powered for the mediation analysis, to explore the possible mediating roles of changes in dietary knowledge, social support, and self-efficacy on the effect of social network measures on weight change, we conducted separate mediation analyses by using structural equation modeling, with changes in each of the theoretical mediators as the mediator and each social network measure as the predictor. In each structural equation modeling model, we controlled for the same covariates as previously mentioned.

## Results

### Participants’ Demographics and Intervention Outcomes

Table 1 presents the baseline characteristics of the participants (N=47) included in the analysis (n=34 for wave I [group 1], n=13 for wave II [group 2]). The mean age of the participants was 46 years with mean baseline BMI of 34.09 kg/m^2^. Participants were predominantly female (44/47, 94%) and mostly African American (32/47, 68%). Almost half (22/47, 47%) reported completing college or graduate school. The baseline characteristics of the participants in the 2 waves/groups were similar and we did not observe noticeable differences in these variables between the waves/groups.

Table 2 reports the change in the main study outcomes during the intervention. Compared with the baseline values, participants experienced an average weight loss of 1.25 kg (*P*=.049), significant increases in dietary knowledge of 2.28 (*P*=.02), and positive dietary social support of 2.87 (*P*<.001), but a nonsignificant decrease in average self-efficacy of 3.07 (*P*=.28).

**Table 1:** Demographics of INSHAPE CLE participants who completed the study.

Characteristics		Group 1 (n=34)	Group 2 (n=13)	Total (N=47)
Age (years), mean (SD)		45.59 (8.91)	48.38 (10.52)	46.36 (9.35)
**Gender, n (%)**				
	Female	31 (91)	13 (100)	44 (94)
	Male	2 (6)	0 (0)	2 (4)
	Transgender	1 (3)	0 (0)	1 (2)
**Race, n (%)**				
	White	9 (27)	3 (23)	12 (26)
	Black or African American	23 (68)	9 (69)	32 (68)
	More than one race	2 (6)	1 (8)	3 (6)
**Education, n (%)**				
	College graduate or more	19 (56)	3 (23)	22 (47)
	Some college	13 (38)	7 (54)	20 (43)
	High school graduate	2 (6)	3 (23)	5 (11)
BMI, mean (SD)		33.79 (3.88)	34.87 (3.86)	34.09 (3.86)

**Table 2:** Study outcomes of INSHAPE CLE participants who completed the study.

Outcome	Baseline values, mean (SD)	Follow-up values, mean (SD)	Mean change (95% CI)	*P* value
Weight (kg)	94.63 (12.85)	93.38 (13.76)	–1.25^a^ (–2.51 to 0.00)	.049
Dietary knowledge (scale 0-100)	27.64 (7.57)	30.08 (8.19)	2.28^a^ (0.44 to 4.13)	.02
Positive dietary social support (scale 5-25)	10.48 (5.27)	13.26 (4.83)	2.87^b^ (1.25 to 4.49)	<.001
Weight loss self-efficacy (scale 0-100)	86.24 (15.30)	83.03 (20.48)	–3.07 (–8.67 to 2.54)	.28

^a^Significant at *P*<.05.

^b^Significant at *P*<.001.

### Social Network Measures and Association With Changes in the Study Outcome

**Figure 2:**
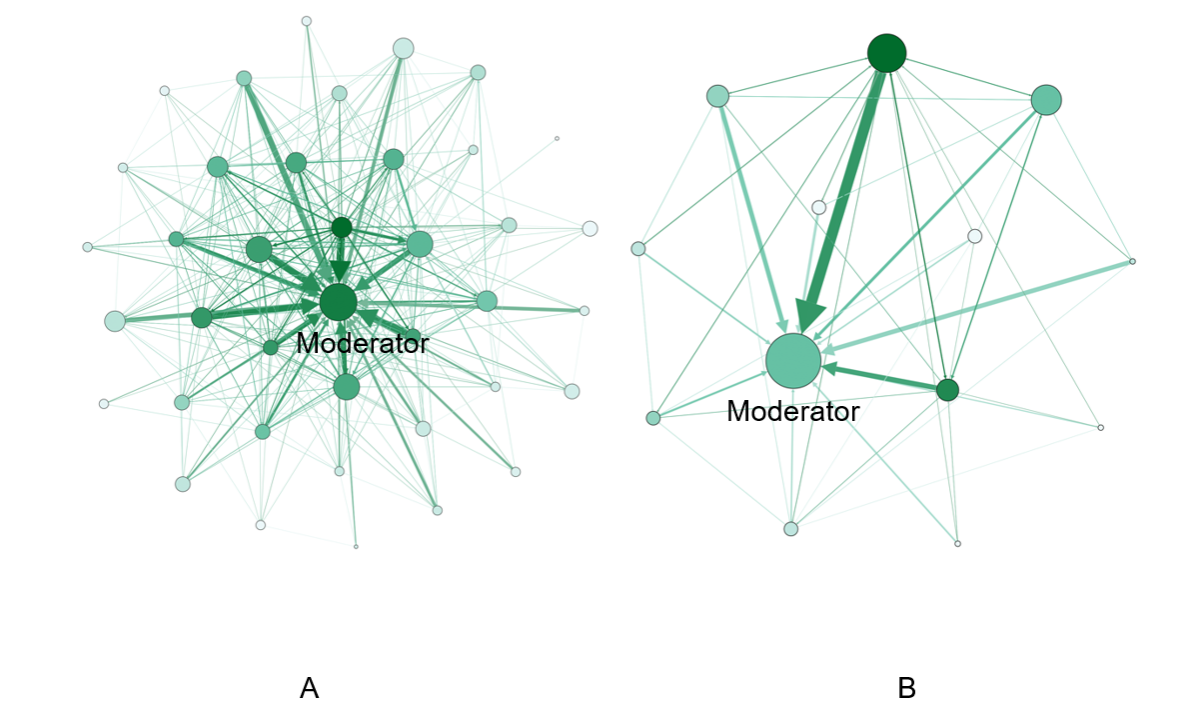
Comment network during the intervention in group 1 (A) and group 2 (B). Link from node 1 to node 2 represents node 1 has made comments to node 2, with thickness representing frequency. Node color represents the number of comments one made to others, with darker color indicating more comments. Node size represents the number of posts one created, with larger size indicating more posts.

Social network was constructed based on participants’ comment relationships during the intervention. Figure 2A and Figure 2B present the comment network among participants in group 1 and group 2, respectively. A link from node 1 to node 2 represents that node 1 has made comments to node 2, with thickness representing frequency. Node color represents the number of comments one made to others, with darker color indicating more comments. Node size represents the number of posts one created, with larger size indicating more posts. Nodes were laid out by a multilevel force-directed algorithm [37]. The network graph intuitively shows there are different levels of engagement among participants—some participants were positioned at the center of the network with many posts or comments, while many participants were positioned at the periphery of the network with very few comments or posts. The structure is more evident in group 1 (where the group size is larger), where k-core analysis [38] showed 14 people formed a core—each person in the core received comments from at least 10 other people in the core. This figure also shows there are different types of engagement among participants—some participants had few original posts but commented on others frequently, some participants did not make many comments to others but received many comments, and some participants were more embedded in the network with their network neighbors tightly connecting to each other. This observation is evident in Table 3, which shows there were large variations in both out-degree (mean 186.32, SD 178.24) and in-degree (mean 25.42, SD 30.83) among the participants. Table 3 also reports the results from regression analyses on how different types of engagement (indicated by social network measures) were associated with changes in study outcomes. Among them, increases in the number of posts, comments, and reactions made (out-degree) significantly predicted weight loss (β=–.94, *P*=.04)—1% increase in the number of posts, comments, and reactions made was associated with 0.0094 kg in weight loss. Increase in the number of comments received (in-degree) significantly predicted increase in self-efficacy (β=7.81, *P*=.009)—1% increase in the number of comments received was associated with 0.0781 units increase in positive self-efficacy change (self-efficacy was measured on a 0-100 scale). In addition, while not significant at .05 level, out-degree was also likely to be positively associated with changes in self-efficacy (β=3.44, *P*=.08), and the degree to which one’s network neighbors are tightly connected with each other (network constraint) was likely to be positively associated with changes in positive dietary social support (β=7.70, *P*=.08). Sensitivity analyses reported in Multimedia Appendix 1 (Table S1 and Table S2) showed that the network structures and the regression results related to in-degree and out-degree were consistent and robust with different ways of constructing networks.

**Table 3:** Associations between social network measures and change in study outcomes for INSHAPE CLE participants.^a^

Predictors/outcome	Mean (SD)	Median (IQR)	Min-Max	Weight change			Dietary knowledge change			Social support change			Self-efficacy change	
				β (95% CI)	*P* value	β (95% CI)		*P* value	β (95% CI)		*P* value	β (95% CI)		*P* value
Out-degree	186.32 (178.24)	127 (238)	1-643	–.94^b^ (–1.85 to –.04)	.04	.06 (–1.13 to 1.24)		.93	.69 (–.37 to 1.75)		.20	3.44^c^ (–.38 to 7.26)		.08
In-degree	25.42 (30.83)	15 (29)	0-174	–.72 (–2.08 to .64)	.29	.94 (–.57 to 2.46)		.21	–.22 (–1.58 to 1.12)		.73	7.81^d^ (2.06 to 13.57)		.009
Network constraint	0.62 (0.24)	0.52 (0.46)	0.23-1.05	–6.16 (–15.99 to 3.66)	.21	–7.78 (–18.36 to 2.79)		.14	7.70^c^ (–.98 to 16.39)		.08	–21.96 (–63.12 to 19.20)		.29

^a^In the analysis, out-degree and in-degree were log-transformed. All models controlled for the outcome before the intervention, the treatment group indicator, age, race, education status, and BMI.

^b^Significant at *P*<.05.

^c^Significant at *P*<.10.

^d^Significant at *P*<.01.

### Mediation Analysis

**Figure 3:**
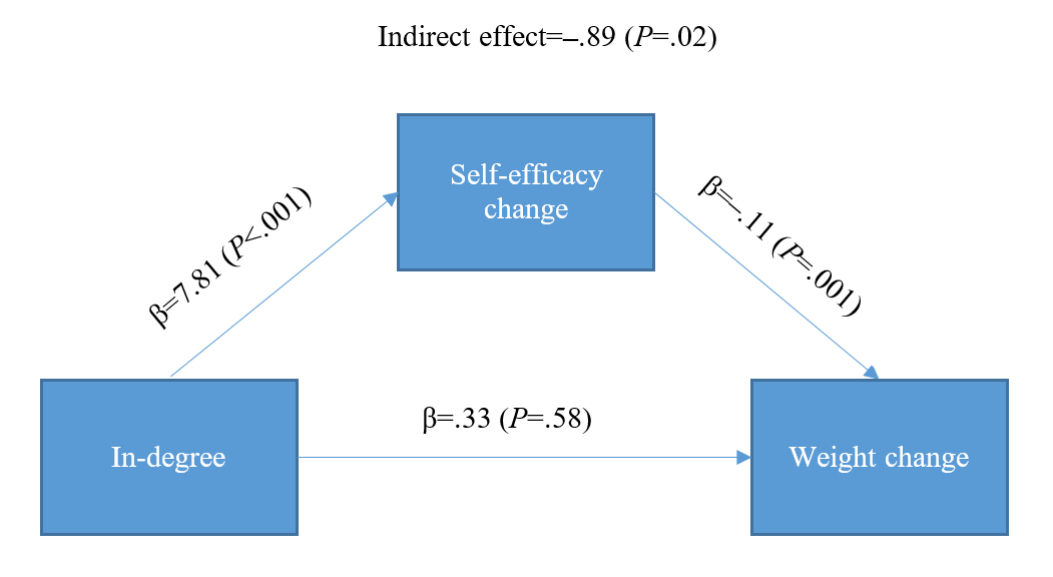
Estimated indirect effect from in-degree to change in self-efficacy to weight loss.

As an exploratory step, we also investigated the possible mediating effects of the theoretical mediators on the effects of social network measures on weight loss, which is presented in Table 4. Structural equation modeling results show that there was a significant indirect effect from in-degree to weight loss that went through a change in self-efficacy (β=–.89, *P*=.02). Figure 3 shows that receiving more comments was positively associated with changes in one’s self-efficacy during the intervention (β=7.81, *P*<.001), which was subsequently associated with more weight loss (β=–.11, *P*=.001).

**Table 4:** Mediation analysis from social network measures to changes in theoretical mediators to weight change for INSHAPE CLE participants.^a^

Predictors/mediators	Dietary knowledge change		Social support change		Self-efficacy change	
	β (95% CI)	*P* value	β (95% CI)	*P* value	β (95% CI)	*P* value
Out-degree	–.01 (–.06 to .05)	.83	–.09 (–.32 to .14)	.43	–.29 (–.64 to .06)	.11
In-degree	–.03 (–.37 to .31)	.88	.04 (–.20 to .29)	.73	–.89^b^ (–1.62 to –.16)	.02
Network constraint	1.22 (–1.52 to 3.95)	.38	–1.21 (–3.84 to 1.40)	.36	2.43 (–1.99 to 6.86)	.28

^a^In the analysis, out-degree and in-degree were log-transformed. All models controlled for the outcome before the intervention, the treatment group indicator, age, race, education status, and BMI.

^b^Significant at *P*<.05.

## Discussion

### Principal Results

In this study, we used social network analysis to analyze the interaction data from a pilot study that delivered a dietary and physical activity intervention to a group of participants of low SES via Facebook. We mapped out participants’ interaction networks over the 12-week intervention period and linked participants’ network characteristics to their behavioral and psychological outcomes such as weight change, self-efficacy, dietary knowledge, as well as perceived social support. Our findings suggest that there is great heterogeneity in the ways and degree to which participants engage in the intervention. Participants engaged at different levels—some participants posted and commented much more than other participants during the intervention, creating a core-periphery structure in the interaction network. Participants also engaged in different ways—some participants had few original posts but commented on others frequently, some participants did not make many comments to others but received many comments, and some participants were more embedded in the network with their network neighbors tightly connecting to each other. Individuals’ network characteristics in the interaction network are predictive of their various study outcomes. Specifically, we found that the number of posts, comments, and reactions one made was directly associated with weight loss, the number of comments one received significantly predicted change in self-efficacy, and the degree to which one’s network neighbors are tightly connected with each other also weakly predicted change in perceived social support. The interactions generated through the intervention may change participants’ weight loss outcomes by affecting their psychological mediators. One mechanism we found was that receiving comments/replies is associated with weight loss through increase in a participant’s self-efficacy.

### Implications

Here, we demonstrate the potential of using social network analysis to understand the social processes of behavior change within the web-based weight loss intervention. Our findings shed light on the social processes participants experienced during the web-based weight loss intervention and how these processes might affect their psychological and behavioral outcomes, which can inform the design of future interventions to achieve better outcomes. For example, the interventionist can monitor the interaction network throughout the intervention and deliberately reach out to those who are less likely to engage. The interventionist could also encourage participants to comment and reply to others more often to boost other participants’ self-efficacy. Moreover, the interventionist could focus on design features to facilitate the formation of cohesive groups, which might increase perceived social support. Finally, the interventionist could train actively engaging participants to be a peer leader who can help with engaging other participants.

### Comparison With Prior Work

While there are ample studies applying the concept of social network analysis in health and health care settings [39-42], not many have been used in the context of web-based behavioral interventions and fewer have specifically focused on weight loss interventions. Our study contributes to this body of literature by improving our understanding of the mechanisms through which social processes generated through intervention affect participants’ psychological and behavioral outcomes. While participant engagement in the Facebook group used in this study exceeded that in previous studies using similar formats targeting diet or physical activity [43,44], similar to previous studies and social media research in other domains, we found great heterogeneity in ways and levels that participants engage in Facebook discussions [45-48]. Consistent with previous studies, we also found that engagement during the intervention was positively associated with weight loss [49,50]. Furthermore, we found that an individual’s various network characteristics are associated with other important psychological outcomes—receiving comments is positively associated with participants’ changes in self-efficacy, and embedding in a more cohesive network (one’s network neighbors more tightly connected with each other) is likely to have a positive association with changes in perceived social support. While not studied in the web-based behavioral intervention context, this is consistent with social network literature from other fields [51,52]. Finally, our mediation analysis shows an interesting path explaining how the intervention might affect participants’ weight loss outcome—receiving comments (in-degree) during the intervention is likely to boost one’s self-efficacy, which resulted in greater weight loss. While the separate links from in-degree to self-efficacy or from self-efficacy to weight loss have been established and validated in other contexts [53,54], to our knowledge, this is the first study to establish this path in web-based behavioral weight loss intervention, which further points to the potential of using social network analysis to understand the mechanisms through which web-based behavioral weight loss intervention affect participants’ psychological and behavioral outcomes.

### Limitations and Future Work

This study has several limitations that point to avenues for future research. First, our sample size was small (N=47) and our participants were predominantly female (44/47, 94%). This limits the statistical power to detect the intervention effects as well as the generalizability of our results to a larger or more gender-balanced population, following a longstanding pattern in weight loss studies of difficulty recruiting male participants [55]. Similarly, we used income as a proxy measure for SES in the inclusion criteria. Given that 47% (22/47) of our participants reported attaining a college degree or advanced degree, we cannot generalize our results to individuals with low levels of education. Future studies should include more male participants and participants with low levels of education to further explore the efficacy of the intervention and how subjects may respond differently. Second, this study did not fully tease out all possible confounding factors and thus cannot establish causality. For instance, participants with higher baseline levels of motivation may be more successful at losing weight and more likely to post comments. Future studies may include more baseline characteristics and utilize randomized controlled designs to better establish causality. Third, in our analysis, the interaction network and associated network measures were aggregated over a 12-week intervention period; thus, we did not fully explore the interaction dynamics during the intervention. Future studies could study how networks change over time and unpack the temporal dynamics between networks and the study outcomes. Fourth, the network in our analysis is primarily constructed from posts and comments and we did not fully explore other relationships such as reactions and views (ie, we did not consider the number of reactions or views one received). While comments and posts have been considered more valuable than other engagement (eg, likes and “lurking”) as they are more cognitively demanding [30], other engagements potentially comprise a substantial proportion of social media use and thus warrant more careful consideration in future studies [56]. Finally, when constructing the interaction network, we did not consider the content of the conversation, which is hypothesized to have different effects on the participants (eg, informational conversation may increase dietary knowledge while emotional support conversation may increase perceived social support). Future studies may employ qualitative analysis and natural language processing to further distinguish the network ties with different content.

### Conclusions

In this pilot study, we constructed participants’ interaction networks by using data from a feasibility trial of a web-based weight loss intervention delivered via Facebook and linked their network characteristics with changes in several important study outcomes of interest such as self-efficacy, social support, and weight. Our results point to the potential of using social network analysis to understand the social processes and mechanisms through which web-based behavioral interventions affect participants’ psychological and behavioral outcomes. Future studies are warranted to validate our results and further explore the relationship between network dynamics and study outcomes in similar and larger trials.

## Appendix

Multimedia Appendix 1 Sensitivity analyses.

## Abbreviations

SES: socioeconomic status
