# Supplementary material for: Social Network Analysis of the Effects of a Social Media–Based Weight Loss Intervention Targeting Adults of Low Socioeconomic Status: Single-Arm Intervention Trial
Source: J Med Internet Res. 2021 Apr 9;23(4):e24690. doi: 10.2196/24690 (PMC8065555; doi:10.2196/24690)
Supplement: Multimedia Appendix 1 [file jmir_v23i4e24690_app1.docx]

**Appendix**

Here we present two sensitivity analyses of the main regression results (in Table 3) based on different methods of constructing networks. (1) We used the original network but calculated out-degree as the total number of comments each individual made during the intervention, and reran the regression analysis using this new measure. Results are presented in the 2^nd^ row in Table S1, which were largely consistent with the results using out-degree based on posts, comments and reactions. (2) We created the comment networks and associated network measures by excluding the moderator-related interactions (i.e. participant-only network), and reran the regression analysis. Figure S1 presents the comment network visualization and Table S2 presents the associated regression results. The results related to in-degree and out-degree were largely consistent with the main results in Table 3 in the main text.

Table S1. Associations between social network measures and change in study outcomes for INSHAPE CLE participants

| Predictors/  Outcome | Mean (SD) | Median [IQR] | Min/Max | Weight change  β [95% CI]  (P-value) | Dietary knowledge change  β [95% CI]  (P-value) | Social support change  β [95% CI]  (P-value) | Self-efficacy change  β [95% CI]  (P-value) |
| --- | --- | --- | --- | --- | --- | --- | --- |
| Out-degree  (Posts+  comments  +reactions) | 186.32 (178.24) | 127  [238] | 1/643 | -.94*  [-1.85 -.04]  (*P*=.042) | .06  [-1.13 1.24]  (*P*=.93) | .69  [-.37 1.75]  (*P*=.20) | 3.44^+^  [-.38 7.26]  (*P*=.076) |
| Out-degree (comments only) | 52.53  (52.08) | 39  [53] | 0/254 | -1.07^+^  [-2.37 .21]  (*P*=.10) | .59  [-1.01 2.19]  (*P*=.46) | .45  [-1.12 2.02]  (*P*=.57) | 5.00*  [.34 9.66]  (*P*=.036) |
| In-degree | 25.42 (30.83) | 15  [29] | 0/174 | -.72  [-2.08 .64]  (*P*=.29) | .94  [-.57 2.46]  (*P*=.21) | -.22  [-1.58 1.12]  (*P*=.73) | 7.81**  [2.06 13.57]  (*P*=.009) |
| Network Constraint | .62  (.24) | .52  [.46] | .23/1.05 | -6.16  [-15.99 3.66]  (*P*=.21) | -7.78  [-18.36 2.79]  (*P*=.14) | 7.70^+^  [-.98 16.39]  (*P*=.080) | -21.96  [-63.12 19.20]  (*P*=.29) |

^+^p<.1; *p<.05; **p<.01; ***p<.001

Note: In the analysis out-degree and in-degree were log-transformed. All models controlled for the outcome before the intervention, the treatment group indicator, age, race, education status and BMI.


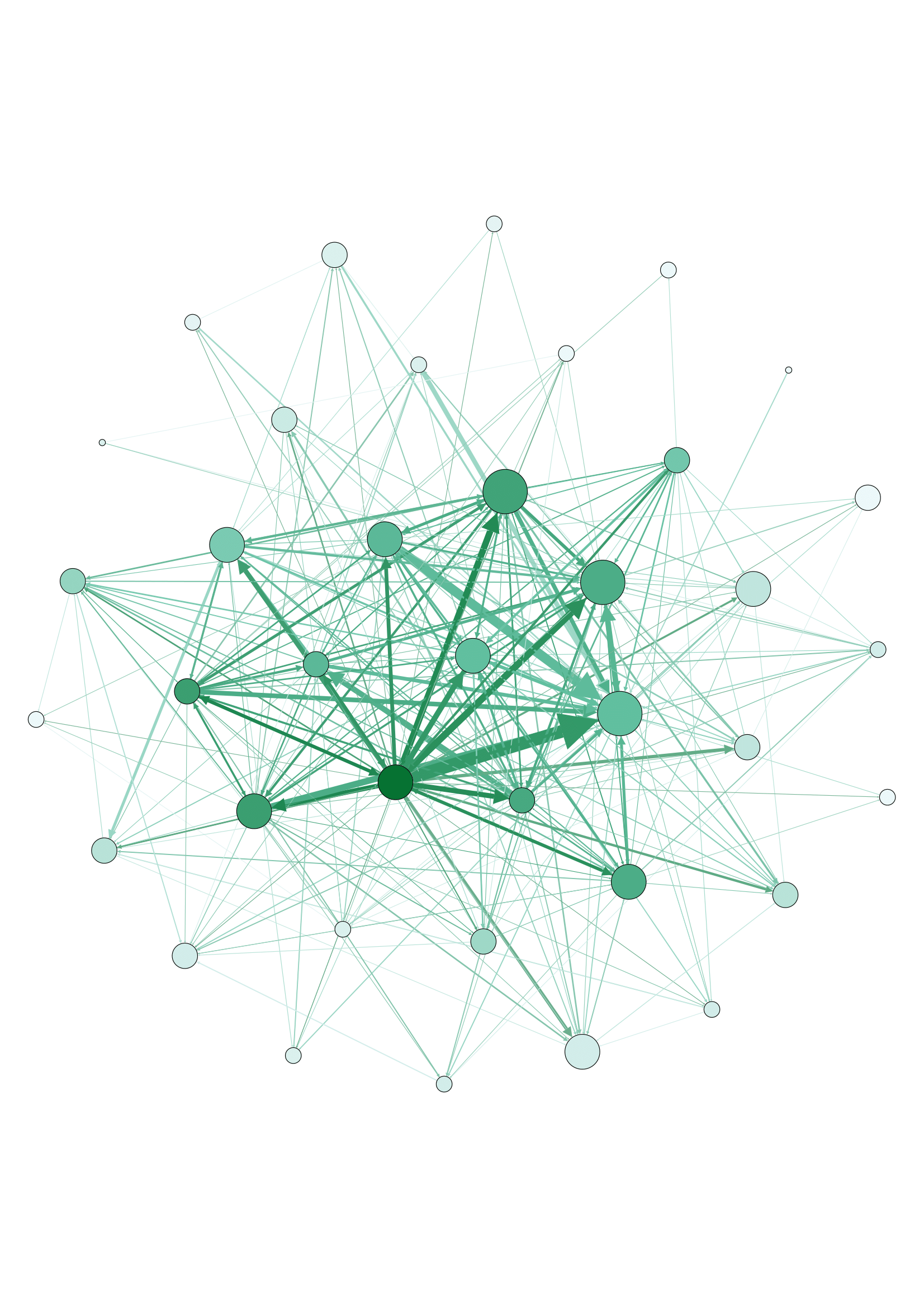

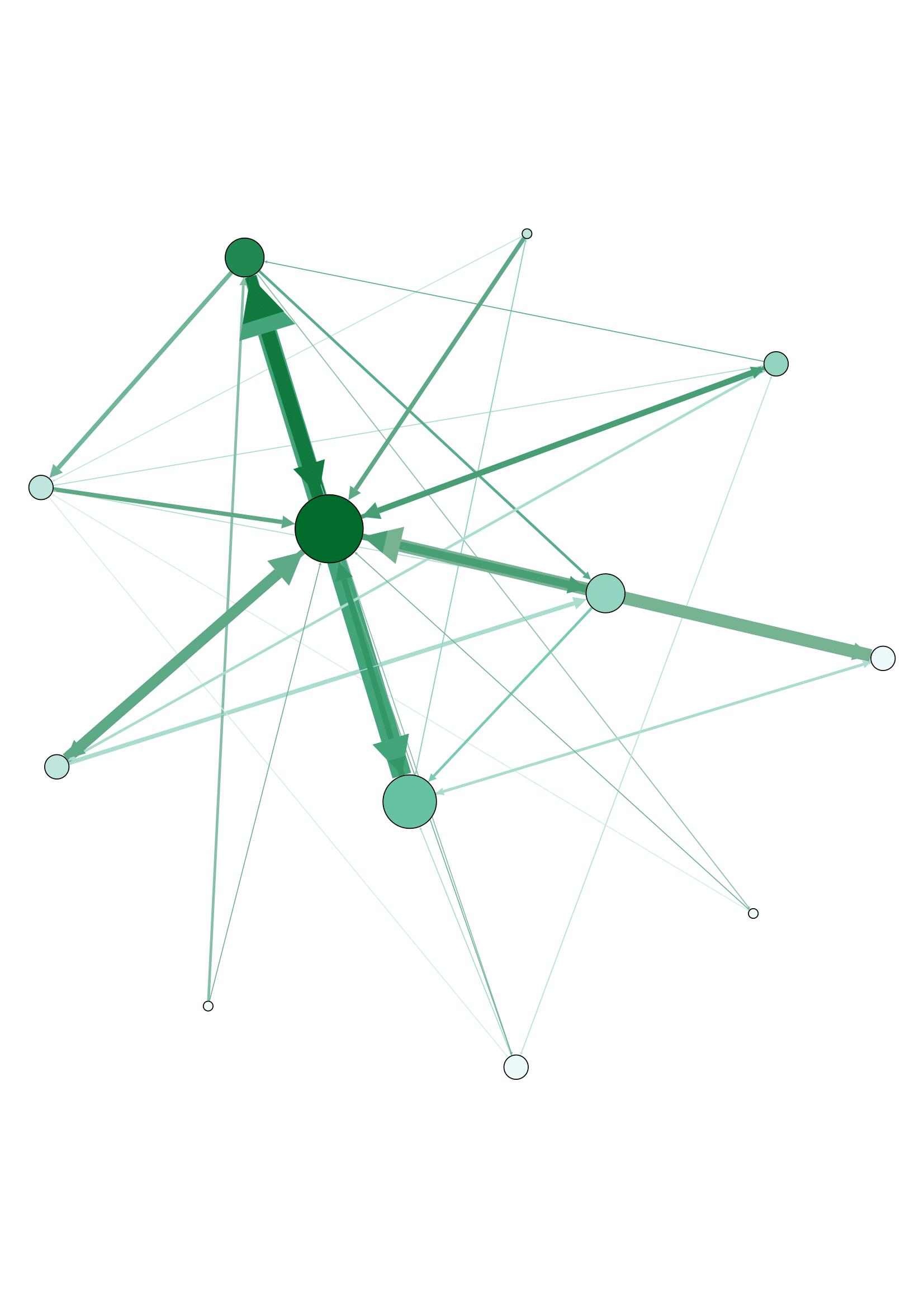


Figure S1. Comment network during the intervention in group 1 (left) and 2 (right) based on participant-to-participant interactions. Link from A to B represents A has made comments to B, with thickness representing frequency. Node color represents the number of comments one made to others, with darker color indicating more comments. Node size represents the number of posts one created, with larger size indicating more posts.

Table S2. Associations between social network measures (based on participant-to-participant interactions) and change in study outcomes for INSHAPE CLE participants

| Predictors/  Outcome | Mean (SD) | Median [IQR] | Min/Max | Weight change  β [95% CI]  (P-value) | Dietary knowledge change  β [95% CI]  (P-value) | Social support change  β [95% CI]  (P-value) | Self-efficacy change  β [95% CI]  (P-value) |
| --- | --- | --- | --- | --- | --- | --- | --- |
| Out-degree | 151.34 (148.39) | 100 [199] | 1/557 | -.99*  [-1.88 -.11]  (*P*=.029) | .14  [-.97 1.25]  (*P*=.80) | .64  [-.44 1.71]  (*P*=.24) | 3.77^+^  [-.03 7.56]  (*P*=.052) |
| In-degree | 22.29 (28.10) | 13  [23] | 0/161 | -.67  [-2.01 .67]  (*P*=.32) | .99  [-.48 2.45]  (*P*=.18) | -.21  [-1.51 1.09]  (*P*=.75) | 7.97**  [3.07 12.87]  (*P*=.002) |
| Network Constraint | .40  [.20] | .30 [.27] | .203/1 | -2.44  [-13.67 8.79]  (*P*=.66) | -12.84*  [-24.52 -1.16]  (*P*=.032) | -4.01  [-14.56 6.54]  (*P*=.45) | -31.27  [-75.56 13.01]  (*P*=.16) |

^+^p<.1; *p<.05; **p<.01; ***p<.001

Note: In the analysis out-degree and in-degree were log-transformed. All models controlled for the outcome before the intervention, the treatment group indicator, age, race, education status and BMI.
